# Reorganization of spatial configurations in visual working memory: A matter of set size?

**DOI:** 10.1371/journal.pone.0225068

**Published:** 2019-11-13

**Authors:** J. David Timm, Frank Papenmeier

**Affiliations:** Department of Psychology, University of Tübingen, Tübingen, Germany; University of Zurich, SWITZERLAND

## Abstract

Humans process single objects in relation to other simultaneously maintained objects in visual working memory. This interdependence is called spatial configuration. Humans are able to reorganize global spatial configurations into relevant partial configurations. We conducted three experiments investigating the process underlying reorganization by manipulating memory set size and the presence of configurations at retrieval. Participants performed a location change detection task for a single object probed at retrieval. At the beginning of each trial, participants memorized the locations of all objects (set size: 4, 8, 12, or 16). During maintenance, a valid retro cue highlighted the side containing the object probed at retrieval, thus enabling participants to reorganize the memorized global spatial configuration to the partial cued configuration. At retrieval, the object probed was shown together with either all objects (complete configuration; Experiment 1a), the cued objects only (congruent configuration; all Experiments), the non-cued objects only (incongruent configuration, all Experiments) or alone (no configuration; Experiment 1b). We observed reorganization of spatial configurations as indicated by a superior location change detection performance with a congruent partial configuration than an incongruent partial configuration across all three experiments. We also observed an overall decrease in accuracy with increasing set size. Most importantly, however, we did not find evidence for a reliable impairment of reorganization with increasing set size. We discuss these findings with regard to the memory representation underlying spatial configurations.

## Background

In everyday life human beings encounter external stimuli, which are memorized as spatial configurations in visual working memory (VWM). In this process, single objects are held in VWM in relation to other simultaneously maintained objects. Examples include driving in traffic, playing video games, participating in sports or watching a movie. This process affects visual information processing and handling of information in VWM. In this study, we focused on the processing of relevant and irrelevant objects stored within a spatial configuration in VWM and the possibility to reorganize such information.

Regarding the organization of VWM, research focuses both on the representation of objects and features in VWM and on the influence of inter-object relations, such as spatial configurations or ensemble statistics, on memory representations. Whereas there is some debate as to whether features, such as color, shape, and location, are stored as integrated objects in VWM [1] or whether features are stored in separate layers and bound by attention [2], research agrees on VWM being a capacity-limited storage subject to narrow limits [3–11]. Further, there is considerable evidence demonstrating the influence of inter-object relations on memory representations, such as memory for single object locations being supported by the global spatial configuration of all objects [12,13] or memory for features of individual objects being biased towards the group mean [14,15]. Less is known, however, about the representation of inter-object relations in memory. In the present research, we investigated the influence of set size on participants’ ability of manipulating the representation underlying inter-object relations during maintenance, more specifically the ability of participants to reorganize a spatial configuration during maintenance based on a retro cue. There are at least two plausible accounts of how inter-object relations might be represented in VWM: either in a manner that is inter-dependent on the objects that they are derived from [16,17] or in a separate and independent storage [18–20]. Those two accounts are particularly interesting, because different predictions regarding the capacity limited nature of the reorganization of inter-object relations can be derived.

If inter-object relations are represented in a manner that is inter-dependent on the objects that they are derived from, increasing set size should result in a reduced fidelity of individual object representations [14,19,21,22] or in the representation of only a subset of individual objects once a capacity limit is exceeded [1], thus also impairing participants ability of reorganizing inter-object relations during memorization with increasing set size. According to accounts suggesting a hierarchical organization of VWM, objects and features are not stored independently but as parts of higher-order representations [14,15,19,23,24]. For example, when observers are asked to memorize the sizes of multiple colored objects, they memorize not only the individual object sizes themselves but also cluster objects according to color, resulting in the recall of individual sizes being biased towards the group mean [14]. Similarly, effects like perceptual grouping influence the memory of single objects’ locations [25]. While one might consider such hierarchical representations as being a case of inter-object relations being represented in a manner that is inter-dependent on the objects they are derived from, studies investigating hierarchical representations focused on the influence of ensembles or configurations on individual objects and leave open how ensembles or configurations might be represented in VWM [19].

If inter-object relations are represented in a separate and independent storage, however, the ability of reorganizing them during memorization might not be subject to set size per se but rather dependent on still to be defined properties of this storage. One account compatible with such a representation is the snapshot account that suggests that inter-object relations such as spatial configurations might be represented as global snapshots in VWM. The core knowledge architecture model of VWM [18], for example, suggests the existence of a specialized storage within VWM storing view-dependent snapshots. Because those snapshots retain the global scene rather than individual objects, they are not subject to the same tight capacity limitations. Previous research showing a viewpoint-dependence of contextual information in VWM supports the idea of view-dependent snapshots in VWM [26]. They found that whereas the manipulation of spatial configurations between encoding and retrieval influenced memory performance for single objects without viewpoint changes, this influence vanished if viewpoint changes were introduced. Further, there is evidence supporting the idea that the global spatial configuration of all encoded objects but not the partial configuration affects memory performance for single objects [12,27] and spatial configurations influence memory performance for single objects also at larger set sizes [12]. Finally, the detection of single object displacements is even impaired if task irrelevant perceptual grouping cues change between encoding and retrieval [25]. Thus, there is research suggesting that inter-object relations, particularly spatial configurations, might be encoded as a global snapshot in VWM.

In our present research, we studied inter-object relations using spatial configurations. In particular, we focused on the influence of set size on the reorganization of spatial configurations in VWM. Reorganization is defined as the process of updating a global spatial configuration into a partial configuration that is cued by a retro cue during maintenance [13]. Our previous results indicate that such a reorganization is possible. In the present study, we asked participants to encode the locations of multiple objects, to maintain those object locations throughout a blank and to detect location change for one probed object during the test. Replicating previous research, location change detection was better with the presence of the complete spatial configuration at retrieval than if no configuration was present [12]. Importantly, we added a retro cue during maintenance. This retro cue indicated the side containing the object for which participants had to perform the location change detection task. Thus, participants had the possibility of updating their memorized global spatial configuration to the configuration congruent to the cue during maintenance. Our results indicate that such a reorganization did occur because location change detection performance for the marked object was superior if the congruent spatial configuration was present than if the incongruent spatial configuration was present. Thus, although only global spatial configurations and not partial spatial configurations were found to support location change detection in previous research [12,27], using a retro cue and presenting the congruent spatial configuration also supported location change detection, indicating that participants reorganized their spatial configuration. This interpretation is also in line with recent research by Bae and Luck [23] who also used a retro cue in a task involving inter-object relations. In their task, participants memorized the orientation of two objects. Whereas they observed interactions between the object orientations (repulsion and attraction) without a cue, introducing a retro cue in their task resulted in the cued object being influenced less by the uncued object. Thus, manipulating attention by means of retro cues modulates the effects of inter-object relations in VWM.

Whereas our previous results showed that the reorganization of spatial configurations is possible, it was less conclusive regarding the influence of set size on this reorganization effect [13]. In particular, whereas the retro cue resulted in reorganization of spatial configurations with a memory set size of six objects in the first experiment, the reorganization effect was not significant with a memory set size of twelve objects in the second experiment. This indicates that the reorganization of spatial configurations might be limited by memory set size. However, the third experiment which was designed to investigate eye-movements, demonstrated reorganization in a free-view condition irrespective of set size.

In the present study, we conducted three new experiments systematically investigating the influence of set size on reorganization of spatial configurations. We hypothesized that reorganization of spatial configurations would be limited by set size. Thus, we predicted that the facilitation caused by the presence of congruent spatial configurations over the incongruent spatial configurations during the test should decrease with increasing memory set size.

## Experiment 1a

With our first two experiments, we investigated the influence of set size on the reorganization of spatial configurations with the four conditions used in our previous research [13]: complete, congruent, incongruent, and no configuration. We split the four possible configurations up into two experiments (Experiment 1a and Experiment 1b) in order to keep the duration of each experiment within one hour per participant.

### Method

We performed the method including sample size, experimental procedure, stimuli characteristics as well as the analysis as we had preregistered on OSF: https://osf.io/r4zvu

#### Participants

We conducted a power analysis using the R-Package powerbydesign [28] with a critical power of .80 at the standard of .05 alpha error probability. The power analysis was designed for a 3 (configuration: complete, congruent, incongruent; within) x 4 (set size: 4, 8, 12, 16; within) repeated-measures ANOVA and we set the bootstrapping iterations to 3000. Based on data from a previous experiment [13], we set the within correlation to *r* = 0.6 and we set the full configuration effect to a difference in d’ of 0.6 (*SD* for each condition: 0.8). Critical to the present manuscript, we ran two versions of the power analysis: one version assuming an interaction of set size and configuration and another version assuming only a main effect of configuration without the respective interaction effect. The critical sample size for observing the interaction in the first power analysis was 28 and for observing the main effect only in the second power analysis was 6. Thus, we recruited 28 participants in order to ensure a power of at least .8 for both possible outcomes, namely the interaction of configuration and set size or a main effect of configuration without the respective interaction. The R code and results for this power analysis are also available online in the OSF project (https://osf.io/tx4u3/).

We preregistered the following exclusion criteria: Participants identified as not performing the task correctly (always pressing the same button or performing at a level that does not deviate from chance) were removed from the data set and replaced by new participants, as well as any participants who did not complete the whole experiment. We had to replace three participants for this particular experiment. Eventually, 28 participants made the final sample in this experiment receiving course credit or monetary compensation of 2€ per 15 min. All of them had normal or corrected-to-normal vision and their age ranged from 19 to 36 years (*M* = 24, *SD* = 3.5). Up to six participants were tested at the same time on different computers. Simultaneous testing and monetary compensation were consistent throughout all experiments. The research was conducted in accordance with APA standards for ethical treatment of participants and with approval of the institutional review board of the University of Tübingen. All participants provided written informed consent.

#### Materials

**Fig 1:**
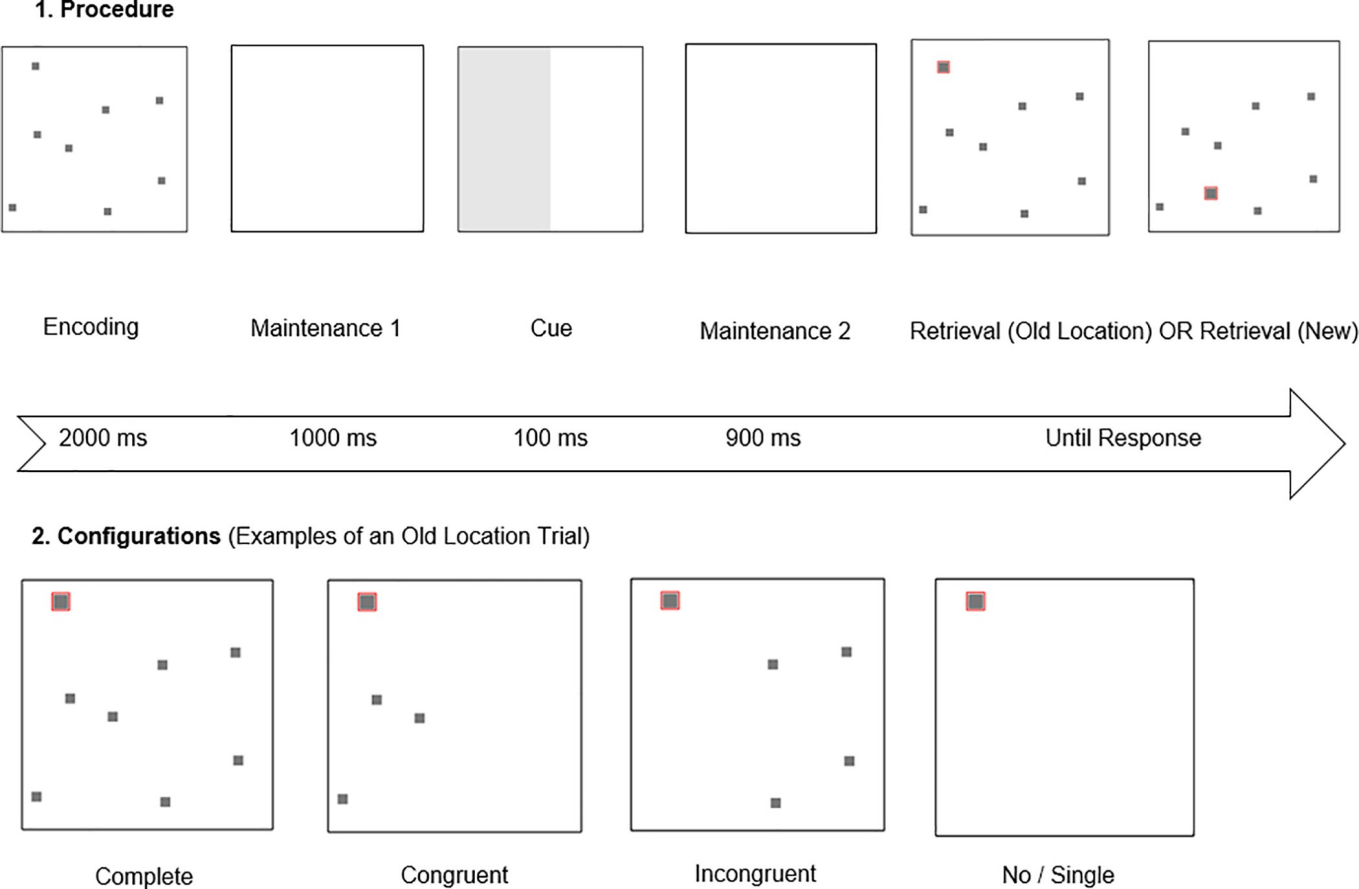
Procedure and manipulations across all experiments. (1) Example trial with all phases and eight objects. Last two panels were intermixed and were used in different trials in each case. (2) Configurations shown in E1a, E1b, or E2. Not every configuration was shown in all experiments. Configurations were intermixed and were used in different trials in each case.

We presented eight grey squares (RGB color hex code: #777777) on a 24” computer screen (Fujitsu Display B24-8 TE Pro) using PsychoPy 1.85.6 [29,30]. Each square measured 1° x 1° (degrees of visual angle). Participants were instructed to fixate a centric cross before each trial. The objects appeared in a 25° x 25° centered array. We did not enforce or measured viewing distance but adjusted the programming code for 40 cm. We generated random object locations for each trial with the same number of squares equally placed on each side, with a minimum center-to-center distance of twice the diameter of a square. Furthermore, this minimum distance was applied to the frame as well as to an invisible vertical center line (Fig 1).

#### Procedure

Participants performed a location change detection task (Fig 1). During encoding all objects were shown for 2000 ms. Afterwards a maintenance phase of 2000 ms followed with no objects shown. At retrieval objects reappeared either in a complete, congruent or incongruent configuration and one object was marked red (RGB color hex code: #FF0000). A grey cue (RGB color hex code: #E6E6E6) was shown 1000 ms into the maintenance period for 100 ms and highlighted the side of the object probed at retrieval. Participants had to press one of two keyboard buttons indicating whether the probed object changed its location or not. On change trials the probed object was presented at a random location within the cued area. It could not appear at a location which was taken by another object at encoding. Moreover, it had the same restrictions as the other objects, for example the minimum inter-object distance. The timing used in our procedure, such as the 2000 ms for encoding, were set to be identical to our previous research [13] because our previous research demonstrated that free-viewing eye-movements might be necessary for configuration effects to occur, despite not yet disentangling during which phase of a trial eye-movements unfold their effect.

We manipulated the cue side (left/right), the set size (4/8/12/16) and the position of the object probed at retrieval (new/old). The cue was 100% valid, so the object probed was always one of the objects indicated by the cue. Moreover, we showed either a complete configuration (presence of object probed and all other objects that were present during encoding), a congruent configuration (presence of object probed and all other objects that were cued) or an incongruent configuration (presence of object probed and all other objects that were not cued) at retrieval.

Trials were presented in randomized order with the restriction that the experiment consisted of four blocks containing 96 trials each, leading to 384 trials in total. Each condition occurred equally often within each block and, thus, also within the whole experiment. Participants performed a practice block, containing one trial per possible condition. The experiment duration was one hour.

#### Analysis

We collapsed data across the factor cue side and compared location change detection performance as indicated by the sensitivity measure across conditions using a 3 (configuration: complete, congruent, incongruent; within) x 4 (set size: 4, 8, 12, 16; within) repeated-measures ANOVA. We calculated sensitivity (according to signal detection theory) as dependent measure across the responses to the old/new probe location trials. We used the sensitivity measurement of d', which is defined as *d*′ = *Φ*^−1^(*phits*)−*Φ*^−1^(*pfa*) with phits being the proportion of hits and pfa being the proportion of false alarms [31]. Hits refer to the accurate detection of old locations, and false alarms refer to responding “old” to a new location. We corrected phits or pfa having values of 0 and 1 with half a trial correct or half a trial incorrect respectively as sensitivity cannot be calculated for those values. Trials with response times exceeding 10 seconds were removed before the analysis (0.18%). When the assumption of sphericity was not met–as indicated by Mauchly’s Test–we corrected the degrees of freedom and p-values with the Greenhouse-Geisser correction. Degrees of freedom accompanying corrected p-values are given with two decimal places. The same analysis was repeated for every experiment.

### Results and discussion

**Fig 2:**
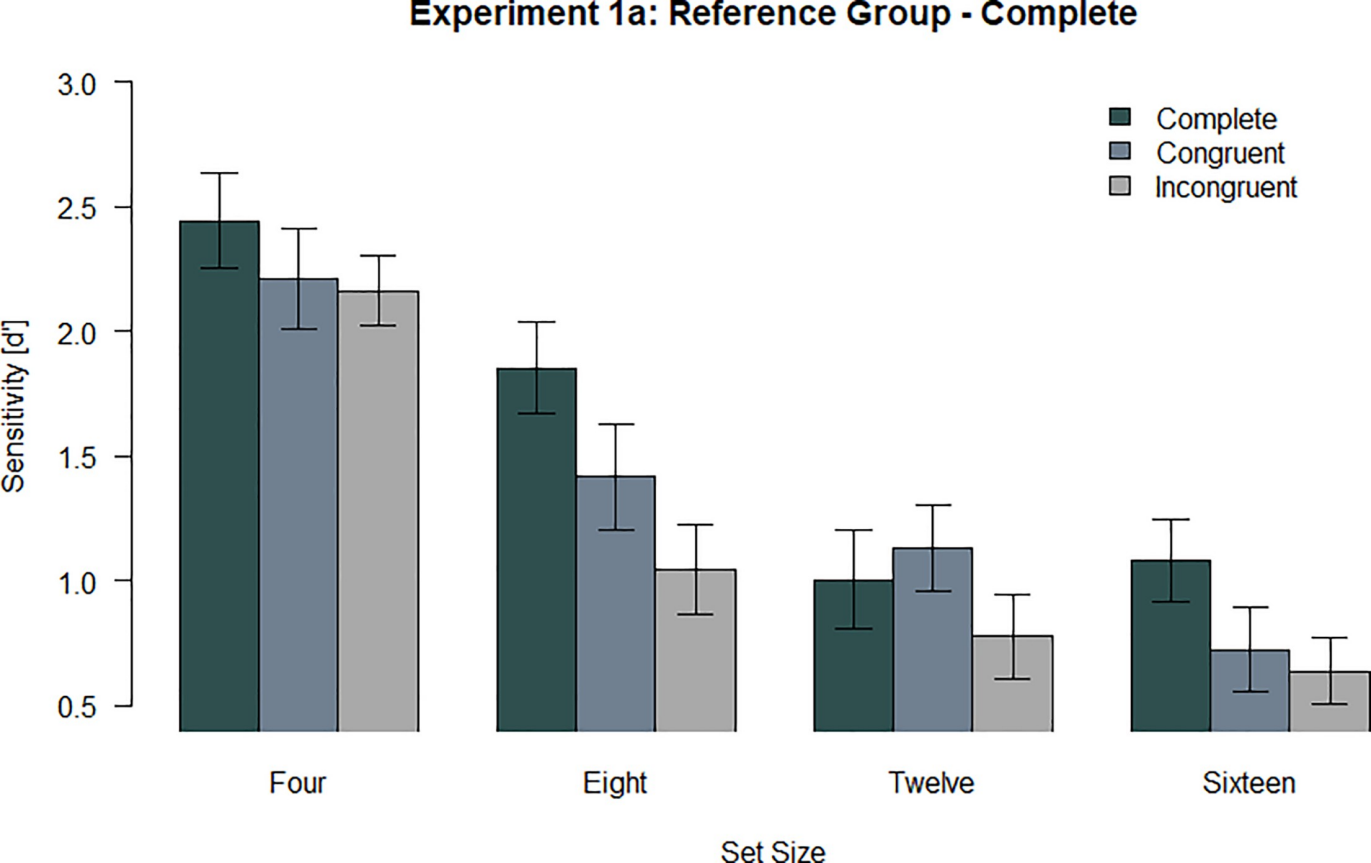
Sensitivity (d’) across all participants for Experiment 1a. Error bars indicate the standard error of the mean (SEM).

All analyses have been preregistered. We hypothesized an interaction effects with the factors configuration and set size. We performed a repeated measures ANOVA with the dependent variable of sensitivity (d’) and the independent variables of set size and spatial configuration. There was a significant main effect for set size, *F* (2.29, 61.94) = 88.24 *p* < .001, η_p_^2^ = .77 and a significant main effect for configuration, *F* (1.63, 43.92) = 26.49, *p* < .001, η_p_^2^ = .50. There was a significant interaction effect, *F* (6, 162) = 2.94, *p* = .010, η_p_^2^ = .10 (Fig 2). We further investigated the interaction effect with t-tests (Table 1). Data and analysis script can be found on OSF: https://osf.io/tx4u3/

**Table 1:** *P-Values–t-tests for configurations in Experiment 1a*.

Set Size	Spatial Configuration	Complete	Congruent
Four	Congruent Incongruent	.062 .049*	.735
Eight	Congruent Incongruent	< .001* < .001*	.021*
Twelve	Congruent Incongruent	.247 .087	.013*
Sixteen	Congruent Incongruent	.013* .001*	.514

* statistically significant (*p <* .05)

We predicted that the reorganization effect vanishes with an increase in set size. When considering set sizes eight, twelve and sixteen, we found evidence regarding this hypothesis: the difference of sensitivity between congruent and incongruent configuration conditions with eight and twelve objects was significant and the effect disappeared with sixteen objects, thus indicating that the reorganization of spatial configuration is affected by set size. This conclusion is further supported by the fact that location change detection performance with a complete configuration was better than the other two configuration conditions at set size sixteen. That is, configuration effects were possible to observe at set size sixteen, but participants did not successfully reorganize spatial configuration containing sixteen objects in VWM.

Surprisingly, we did not observe a significant reorganization effect at set size four. We can only speculate about the source of this unexpected finding. One the one hand, this might be caused by a cost-benefit consideration: four objects can be easily held in VWM such that participants might not invest the effort to reorganize the spatial configuration in VWM based on the cue given during maintenance. On the other hand, the congruent configuration consists of only two objects at set size four: the object probed and one other object on the same side of the display. Possibly, two objects do not form a spatial configuration that can draw on the memory representation used to represent spatial configurations, thus not enabling participants to reorganize the original configuration containing four objects to a congruent configuration containing only two objects.

## Experiment 1b

This experiment was similar to Experiment 1a. We replaced the complete configuration condition with a no configuration condition.

### Method

We performed the method including sample size, experimental procedure, stimuli characteristics as well as the analysis as we had preregistered on OSF: https://osf.io/y4npj

#### Participants

We used the same sample size metrics as in Experiment 1a. Subsequently, 28 participants were invited for this experiment receiving course credit or monetary compensation. We applied the same exclusion criteria and seven participants had to be replaced due to a performance level that did not deviate from chance leading to the participation of 35 subjects but a final sample of 28 as previously appointed. Participants of the final sample had normal or corrected-to-normal vision and their age ranged from 21 to 30 years (*M* = 24.5, *SD* = 2.1). All participants provided written informed consent.

#### Materials

We used the same materials and setup as in Experiment 1a.

#### Procedure

We used a similar procedure as in Experiment 1a but replaced the complete configuration with the no configuration condition. In detail, we showed a single object condition (presence of object probed only) in this experiment, a congruent configuration (presence of object probed and all other objects that were cued) and an incongruent configuration (presence of object probed and all other objects that were not cued) at retrieval.

#### Analysis

The analyses were consistent with analyses described above. We collapsed data across the factor cue side and compared location change detection performance as indicated by the sensitivity measure across conditions using a 3 (configuration: single, congruent, incongruent; within) x 4 (set size: 4, 8, 12, 16; within) repeated-measures ANOVA. We calculated sensitivity (according to signal detection theory) as dependent measure across the responses to the old/new probe location trials. Trials with response times exceeding 10 seconds were removed before the analysis (0.03%).

### Results and discussion

**Fig 3:**
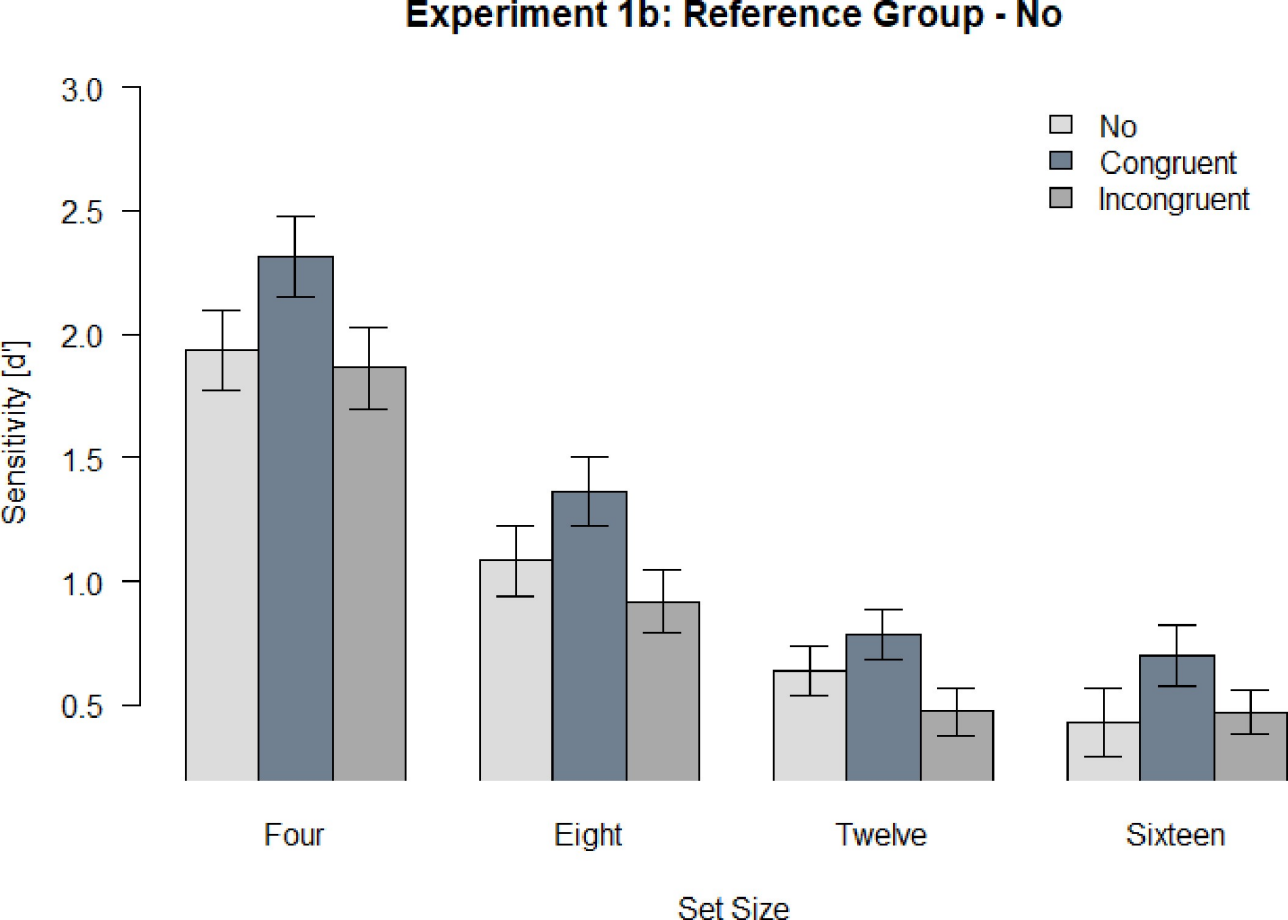
Sensitivity (d’) across all participants for Experiment 1b. Error bars indicate the standard error of the mean (SEM).

All analyses have been preregistered. We hypothesized an interaction effect of the factors configuration and set size. We performed a repeated measures ANOVA with the dependent variable of sensitivity (d’) and the independent variables of set size and spatial configuration. There was a significant main effect for set size, *F* (2.34, 63.18) = 112.77 *p* < .001, η_p_^2^ = .81 and a significant main effect for configuration, *F* (2, 54) = 14.14, *p* < .001, η_p_^2^ = .34. There was no significant interaction effect, *F* (6, 162) = 0.68, *p* = .663, η_p_^2^ = .02 (Fig 3). We further investigated the main effect of configuration with t-tests (Table 2). Data and analysis script can be found on OSF: https://osf.io/r2zah/

**Table 2:** *P-Values–t-tests for configurations in Experiment 1b*.

Set Size	Spatial Configurations	No	Incongruent
Aggregated	Congruent Incongruent	.002* .217	< .001*

* statistically significant (p < .05)

As in Experiment 1a, we again predicted that the reorganization effect vanishes with an increase in set size. Surprisingly, however, we did not observe the predicted interaction effect. Instead, there was a main effect of configuration indicating that the reorganization of spatial configuration in VWM was unaffected by set size. That is, the difference of sensitivity between congruent and incongruent configuration conditions was significant independent of set size. In order to further investigate this contradictory finding, we conducted Experiment 2.

## Experiment 2

With our Experiments 1a and 1b, we investigated the influence of set size on the reorganization of spatial configurations in VWM. Whereas we observed the predicted interaction of set size and configuration in Experiment 1a, this interaction was not significant in Experiment 1b. We interpreted the difference in location change detection performance between the congruent and incongruent configuration conditions as the critical comparison indicating the occurrence of reorganization. We also presented two different reference conditions across Experiments 1a and 1b. The no configuration (Experiment 1b) served as reference condition indicating whether the configuration effect was present with the reorganized configuration leading to a superior location change detection performance with a congruent than no configuration. The complete configuration (Experiment 1a) served as reference condition indicating whether the reorganization occurred in full or not. This was shown with the difference in location change detection performance between the complete configuration condition and congruent configuration condition. With the present experiment, we wanted to further investigate the reorganization effect in terms of the capacity limit and as another measure regarding our contradictory findings in the previous two experiments. In particular, we were interested in the difference in performance between the congruent and incongruent conditions in the absence of reference conditions because their addition in Experiments 1a and 1b could have affected the processing of the displays, for example by implementing different encoding or maintenance strategies such as focusing more or less on configurational information due to the presence of the complete configuration in contrast to the single item condition. Thus, we abandoned both the complete configuration and the no configuration condition in this experiment and focused solely on the comparison of the congruent and incongruent configurations across set size.

### Method

We performed the method including sample size, experimental procedure, stimuli characteristics as well as the analysis as we had preregistered on OSF: https://osf.io/xndkc

#### Participants

We used the same sample size metrics and exclusion criteria as before. Two participants had to be replaced due to a performance level that did not deviate from chance leading to the participation of 30 subjects but a final sample of 28 as previously appointed. Participants of the final sample had normal or corrected-to-normal vision and their age ranged from 20 to 34 years (*M* = 24, *SD* = 3.2). All participants provided written informed consent.

#### Materials

We used the same materials and setup as in Experiments 1a and 1b for the location change detection task.

#### Procedure

We used a similar procedure as in Experiments 1a and 1b. In contrast, we only showed a congruent configuration (presence of object probed and all other objects that were cued) or incongruent configuration (presence of object probed and all other objects that were not cued) at retrieval in this experiment.

#### Analysis

The analyses were consistent with analyses described above. We collapsed data across the factor cue side and compared location change detection performance as indicated by the sensitivity measure across conditions using a 2 (configuration: congruent, incongruent; within) x 4 (set size: 4, 8, 12, 16; within) repeated-measures ANOVA. We calculated sensitivity (according to signal detection theory) as dependent measure across the responses to the old/new probe location trials. Trials with response times exceeding 10 seconds were removed before the analysis (0.01%).

### Results and discussion

**Fig 4:**
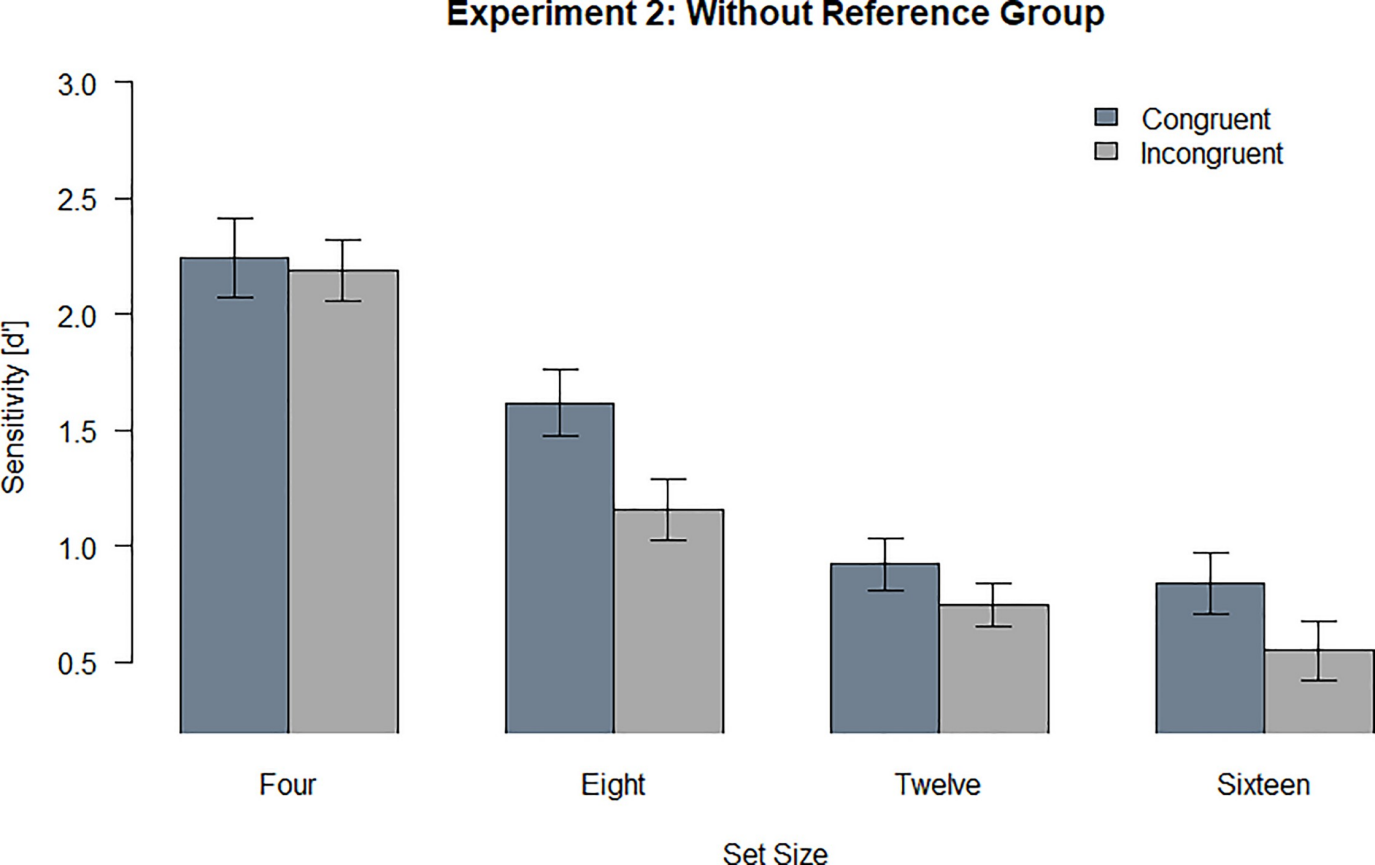
Sensitivity (d’) across all participants for Experiment 2. Error bars indicate the standard error of the mean (SEM).

All analyses have been preregistered. We performed a repeated measures ANOVA with the dependent variable of sensitivity (d’) and the independent variables of set size and spatial configuration. There was a significant main effect for set size, *F* (3, 81) = 79.15 *p* < .001, η_p_^2^ = .75 and a significant main effect for configuration, *F* (1,27) = 13.51, *p* = .001, η_p_^2^ = .33. There was no significant interaction effect, *F* (3, 81) = 2.26, *p* = .088, η_p_^2^ = .08 (Fig 4). Data and analysis script can be found on OSF: https://osf.io/8ruxn/

Once again, we observed no significant interaction effect of set size and configuration but only the two main effects of set size and configuration. The location change detection performance was superior in the congruent configuration than in the incongruent configuration up to sixteen objects. Therefore, the results indicate that the reorganization effect is largely unaffected by set size and the reorganization effect did not vanish with an increase in set size. Despite the non-significant interaction, the descriptive results also indicate that the occurrence of reorganization at set size four might not be reliable, which is similar to what we observed in Experiment 1a.

## Cross-experiment analysis

Whereas we found an interaction effect of set size and configuration in Experiment 1a as we had predicted beforehand, we could not find this interaction effect in Experiments 1b and 2. In order to further investigate the contradictory findings regarding the influence of set size on reorganization across our experiments, we calculated a cross-experiment analysis across our three experiments. For this analysis, we used the data of the congruent and incongruent configuration conditions as they occurred in all three experiments and calculated a 2 (configuration: congruent, incongruent; within) x 4 (set size: 4, 8, 12, 16; within) x 3 (experiment: 1a, 1b, 2; between) ANOVA. Similar to Experiments 1b and 2, there were significant main effects for set size and configuration, both *p*s < .001, and a non-significant interaction of set size and configuration, *F* (3, 243) = 2.39, *p* = .069, η_p_^2^ = .03. Importantly, neither the main effect for experiment nor connected interaction effects were statistically significant, *p*s between .277 and .556. In order to quantify the evidence for the absence of the interaction of set size and configuration, we calculated the Bayes factor comparing a basic model (H0) including the main effects for set size and configuration only to a full model (H1) including both main effects and the corresponding interaction of set size and configuration. Both models included “participant” as a random effect. The Bayes factor was *BF*10 = 0.13 suggesting that our data provides evidence for the absence rather than presence of the interaction of set size and configuration, which is in accordance with our ANOVA results. Thus, there was no reliable influence of set size on the reorganization of spatial configurations in VWM across our experiments. The analysis script can be found on OSF: https://osf.io/8ruxn/

In a nutshell, two of our experiments (1b/2) and a cross-experiment analysis with a high number of participants could not provide evidence that the reorganization effect vanishes with an increase in set size. Thus, we conclude that reorganization of spatial configuration is largely unaffected by set size, at least within the range of set sizes investigated, that is up to sixteen objects.

## General discussion

We conducted three experiments to investigate whether reorganization of spatial configurations in VWM is affected by set size. A valid retro cue presented during maintenance enabled participants to reorganize their spatial configuration to the cued side of the display. We measured reorganization by comparing location change detection performance of a single probed object under the presence of either the congruent (all cued objects) or incongruent (probed object and all not-cued objects) configuration. Whereas reorganization of configurations was affected by set size in Experiment 1a –reorganization of configurations at set sizes eight and twelve but not sixteen–no significant influence of set size on reorganization was found in Experiments 1b and 2 and in a cross-experiment analysis. We conclude that reorganization of spatial configurations is largely unaffected by set size.

Previous research suggested a slot model for VWM with a fixed maximum capacity threshold [1,3–11]. In the present research, we investigated the influence of set size on the participants’ ability of manipulating the representation underlying inter-object relations during maintenance, more specific the ability of participants to reorganize a spatial configuration during maintenance based on a retro cue. There are at least two plausible accounts of how inter-object relations might be represented in VWM: either in a manner that is inter-dependent on the objects that they are derived from or in a separate and independent storage [18,19]. While one would expect a strong influence of set size on the reorganization of spatial configurations in VWM based on the former account, a much weaker influence of set size on the reorganization of spatial configurations would be expected based on the latter account. Our results speak in favor of the latter account and thus an independent storage for spatial configurations in VWM as set size did not lead to reliable reduction of the reorganization effect across the set sizes investigated in our experiments. One potential candidate for this independent storage is the view-dependent snapshot storage proposed by the core knowledge architecture model of VWM [18]. Given our general finding that reorganization is possible, however, this storage cannot be formalized as holding representations of spatial configurations as a frozen or rigid snapshot in VWM [18,26,32] but rather as a flexible storage within a global snapshot approach or alternatively as part of a flexible hierarchical representation. Future research could also establish whether there is a potential relation between this independent storage for configurations and the high-capacity fragile VWM store proposed by previous research [33], for example by manipulating the presence of backward-masks after encoding.

We concluded that set size does not affect reorganization of spatial configurations. This is limited in some points. For example, with four objects this effect was only shown in Experiment 1b. As already argued, reorganization might not be useful in this instance as the whole configuration of four objects is already easy to remember and reorganization seems ineffective. So that might be the threshold, where single objects relegate into a spatial configuration maintained in VWM caused by a cost-benefit consideration. Furthermore, the congruent configuration consists of only two objects at set size four: the object probed and one other object on the same side of the display. Possibly, two objects do not form a spatial configuration that can draw on the memory representation used to represent spatial configurations, thus not enabling participants to reorganize the original configuration containing four objects to a congruent configuration containing only two objects. Recent research suggested, that items were not bound to spatial configuration representations in VWM. Interestingly, the configurations in all these experiments consisted of no more than two items and no configuration effect was found [34]. This stands in line with the majority of our experimental results that too few objects might not form a stable spatial configuration. On the other hand, the reorganization of spatial configurations with four objects was observed in Experiment 1b. Therefore, the minimum threshold for the possibility of reorganization of spatial configurations cannot be established yet and should be investigated in future research.

We defined the partial configurations as congruent and incongruent, which apply to the relevant and irrelevant side of the screen. One might argue, that relevant and irrelevant items are also the objects near and far to the probed object. In our design, we randomized locations of the objects and therefore, also incongruent objects (e.g., next to the center) could be closer to the object probed than a congruent object (e.g., placed in the corner of the display). By analyzing a number of random locations generated by our location generation algorithm, we could confirm that the close items were not identical to the congruent configuration in our experiments. For example, with a set size of eight objects, we picked the three nearest objects (because a congruent configuration consisted of the object probed and three additional objects) and had a look if those three closest objects originated from the congruent configuration only or whether they also included objects from the incongruent configuration. Indeed, on about 2/3 of the trials at least one (and up to three) incongruent objects were part of the three objects closest to the object probed. Therefore, the objects closest to the probed object did not consist of the congruent objects only and thus, distance and congruence/relevance were not the same. Further, the ratio of the average distance between the target and the congruent items and the average distance between the target and the incongruent items was comparable across set size conditions. In previous studies, also the intact configuration rather than absolute location or distance of objects was shown to be important [12]. This is not to say that distance does not play a role for configurational processing at all. Importantly, however, we are confident that our congruent vs. incongruent findings cannot be explained by a pure distance account. Nevertheless, future research should systematically investigate the role of distance and object prioritization by cues on configurational processing and reorganization.

Another point to discuss are the configurations used as reference (complete & no) we used in our experiments. In Experiment 1a, the levels of location change detection performance in the complete configuration were better than the incongruent configuration. The performance levels in the complete configuration was superior than or equal to the congruent configuration levels. This also speaks for a successful reorganization as the location change detection performance level of the congruent configuration should be similar to the performance level in the complete configuration, while the level of the incongruent configuration should be inferior than the performance levels of the complete or congruent configuration to infer the reorganization effect. In Experiment 1b, the levels of location change detection performance in the no configuration condition were worse than in the congruent configuration. The performance level in the no configuration was equal to the incongruent configuration level. This favors a successfully evoked configuration effect. Location change detection should be higher with a surrounding configuration than with a single object only as a configuration supports memory representation–this was shown with the congruent vs. no comparison. Nevertheless, a configuration effect needs relevant objects, and irrelevant ones can impair memory recognition compared to congruent configurations–this was shown with the incongruent vs. no comparison, which were equal. So, with both experiments and reference conditions, we find support for both successful configuration memory advantages and the accompanying possibility of reorganization of spatial configurations. Nevertheless, we found different patterns of reorganization when we compared Experiments 1a and 1b using different reference conditions which might affected the extent of reorganization of spatial configurations. In Experiment 1a, we found a significant interaction effect while we did not find one in Experiment 1b. So, one might argue that the reference conditions might have affect the reorganization effect, but our cross-experiment analysis showed that across all three experiments there was no interaction effect regarding the reference conditions. So, reorganization as a difference of congruent and incongruent configuration memory performance was independent of reference conditions used. All in all, a conclusion of an effect of the reference conditions on the reorganization of spatial configurations cannot be drawn from our experimental results.

Concluding, reorganization of spatial configurations from a global and complete configuration into relevant (here congruent) and irrelevant (here incongruent) partial configurations was unaffected by set size. This speaks in favor of models suggesting the representation of configurations in a separate and independent storage, that is not subject to the same tight capacity limits suggested by classical VWM slot models. Future research can use our results as a starting point when the reorganization effect is investigated. There are several points that are still unclear, for example, if and how the cue and the different configurations are used. In a nutshell, we observed reorganization of spatial configurations as indicated by a superior location change detection performance with a congruent partial configuration than an incongruent partial configuration across all three experiments. We conclude that besides the overall decrease in accuracy the reorganization effect was stable across all experiments and that the reorganization effect itself was largely unaffected by any of the set sizes investigated.
